# Changes in Malaria Parasite Drug Resistance in an Endemic Population Over a 25-Year Period With Resulting Genomic Evidence of Selection

**DOI:** 10.1093/infdis/jit618

**Published:** 2013-11-21

**Authors:** Davis C. Nwakanma, Craig W. Duffy, Alfred Amambua-Ngwa, Eniyou C. Oriero, Kalifa A. Bojang, Margaret Pinder, Chris J. Drakeley, Colin J. Sutherland, Paul J. Milligan, Bronwyn MacInnis, Dominic P. Kwiatkowski, Taane G. Clark, Brian M. Greenwood, David J. Conway

**Affiliations:** 1 Medical Research Council Unit, Fajara, The Gambia; 2 London School of Hygiene and Tropical Medicine, London; 3 Wellcome Trust Sanger Institute, Hinxton, Cambridge; 4 Medical Research Council Centre for Genomics and Global Health, and Wellcome Trust Centre for Human Genetics, University of Oxford, United Kingdom

**Keywords:** resistance monitoring, population genetics, genomic surveillance, archival analysis, policy and practice

## Abstract

***Background.*** Analysis of genome-wide polymorphism in many organisms has potential to identify genes under recent selection. However, data on historical allele frequency changes are rarely available for direct confirmation.

***Methods.*** We genotyped single nucleotide polymorphisms (SNPs) in 4 *Plasmodium falciparum* drug resistance genes in 668 archived parasite-positive blood samples of a Gambian population between 1984 and 2008. This covered a period before antimalarial resistance was detected locally, through subsequent failure of multiple drugs until introduction of artemisinin combination therapy. We separately performed genome-wide sequence analysis of 52 clinical isolates from 2008 to prospect for loci under recent directional selection.

***Results.*** Resistance alleles increased from very low frequencies, peaking in 2000 for chloroquine resistance-associated *crt* and *mdr1* genes and at the end of the survey period for *dhfr* and *dhps* genes respectively associated with pyrimethamine and sulfadoxine resistance. Temporal changes fit a model incorporating likely selection coefficients over the period. Three of the drug resistance loci were in the top 4 regions under strong selection implicated by the genome-wide analysis.

***Conclusions.*** Genome-wide polymorphism analysis of an endemic population sample robustly identifies loci with detailed documentation of recent selection, demonstrating power to prospectively detect emerging drug resistance genes.

An understanding of the adaptation of pathogens to changing environments and specific control efforts is important but challenging; however, developments in genomic methods offer approaches that may become widely effective. When new alleles are strongly selected within a population, associated haplotypes in flanking regions of the genome affect local linkage disequilibrium and the allele frequency spectrum [1]. However, evaluation of such statistical signatures against evidence of historical selection has rarely been performed directly, as estimates obtained by measuring changes in allele frequencies over time are rarely possible for natural populations.

In the human malaria parasite *Plasmodium falciparum*, alleles have been widely surveyed for genes that determine resistance to chloroquine (*crt* on chromosome 7 encoding the chloroquine resistance transporter and *mdr1* on chromosome 5 encoding a multidrug resistance transporter) or antifolates (*dhfr* on chromosome 4 encoding the target of pyrimethamine and *dhps* on chromosome 8 encoding the target of sulfadoxine). Particular haplotypes at each of these chromosomal loci have undergone selective sweeps [2–7], some of these spreading from Asia into Africa [2, 8–10]. Several studies have compared proportions of alleles in endemic populations at different times, for example, in Mozambique over 5 years [11], Tanzania over 6 years [12], western Kenya over 8 years [13], Malawi over 8 years [14] and 9 years [15], Papua New Guinea over 12 years [16], Gabon over 14 years [17], and eastern Kenya over 14 years [18]. In each case, resistance alleles were already common at the start of the survey period. In addition to positive selection by drugs, there has also been a decline in prevalence of chloroquine resistance alleles after use of the drug ended in particular populations [14, 19], highlighting the existence of fitness costs and suggesting the potential for future reintroduction of chloroquine [20]. However, each of these temporal comparisons covered only part of the period during which local changes in drug resistance occurred, and none of them were directly related to attempts to detect or interpret signatures of selection from genome-wide analyses.

Here, we report the longest temporal survey of drug resistance genes within an endemic population, covering different phases of antimalarial drug use and focusing on the 4 gene loci in *P. falciparum* that are responsible for chloroquine resistance (*crt*, *mdr1*) and antifolate resistance (*dhfr*, *dhps*). Parasite samples were collected in The Gambia over a 25-year period from 1984, when resistance was unknown locally, through the subsequent gradual failure of chloroquine and antifolate therapy, until the eventual switch to artemisinin combination therapy in 2008. The changes in allele frequencies are related to therapeutic policies and practices at different times during the period. We then surveyed whole parasite genome sequences from an independent sample of clinical isolates at the end of this period and examined the statistical impact of selection on these loci compared with the rest of the genome that encodes more than 5000 other genes. Results highlight the accuracy of genomic signatures of selection, as well as their transient nature, and identify additional loci under recent strong selection, indicating the need to apply these approaches more intensively in Africa where the public health implications of the spread of drug resistance are most profound.

## METHODS

### Ethics Statement

The Joint Ethics Committee (JEC) of the Gambia Government (JEC) and Medical Research Council Gambia Unit (MRC) approved this study. All subjects gave informed consent for sample collection, and procedures were conducted in accordance with the principles expressed in the Declaration of Helsinki.

### Retrospective Sampling for *P. falciparum* Drug Resistance Polymorphisms

Blood samples from subjects with *P. falciparum* malaria infections living in the Farafenni area of the North Bank Region of The Gambia in West Africa were collected during 8 different years from 1984 to 2008. Sample collection was part of previous studies conducted by the MRC Gambia Unit that were approved by the JEC. Samples were stored in the MRC Gambia laboratories as aliquots of heparinized blood frozen at −20°C or as dried blood spots sealed with desiccant at ambient temperature. The identification and extraction of stored samples for this genotyping study were reviewed and approved by the JEC. DNA extraction from each sample was performed using the QIAamp DNA mini kit, and species-specific polymerase chain reaction (PCR) was performed to identify samples with *P. falciparum* DNA. This screening identified 668 blood samples with *P. falciparum* DNA available for genotypic analysis. The samples came from individuals who had not received antimalarial treatment during their infection prior to sampling: 132 in 1984, 102 in 1988, 66 in 1991, 87 in 1998, 97 in 2000, 70 in 2004, 78 in 2007, and 36 in 2008. The origins and summary details of the sample sets are given in Table 1.

**Table 1.** Sources and Summary Details of Blood Samples Collected Between 1984 and 2008 Used for *Plasmodium falciparum* Genotyping

Year	Time of Sampling	Age Group	Type of Sampling and Clinical Status	Previous Description of Sampling	Number of Samples Genotyped
1984	August	All ages	Community, asymptomatic	[21]	132
1988	May	3–8 y	Community, asymptomatic	[22]	102
1991	June and November	All ages	Community, asymptomatic	[23]	66
1998	September–December	<15 y	Health center, uncomplicated malaria	[24]	87
2000	June–November	All ages	Community, asymptomatic	Unpublished	97
2004	October–December	<10 y	Health center, uncomplicated malaria	[25]	70
2007	November	All ages	Hospital, uncomplicated malaria	[26]	78
2008	August–December	<15 y	Community, asymptomatic	[27]	36

### *P. falciparum* Drug Resistance Genotyping

The following 4 *P. falciparum* drug resistance loci were genotyped by restriction fragment length polymorphism analysis of PCR products on ethidium bromide–stained agarose gels: *dhfr* gene codons 51, 59, and 108 (chromosome 4, gene ID PF3D7_0417200); *mdr1* gene codon 86 (chromosome 5, gene ID PF3D7_0523000); *crt* gene codon 76 (chromosome 7, gene ID PF3D7_0709000); and *dhps* gene codons 437 and 540 (chromosome 8, gene ID PF3D7_0810800). This was done using previously described methods [28–30].

### Analysis of Temporal Changes in Allele Frequencies

Proportions of isolates containing resistance alleles (either alone or with sensitive alleles in the case of mixed genotype infections) were compared between successive sample years, and significance was assessed using Fisher's exact test. Then, for selection modeling, allele frequencies in each year sampled were estimated by counting 1 allele per isolate, randomly sampling from mixed genotype isolates; 95% confidence intervals (CIs) were determined using the modified Wald method. We made simplified assumptions of 2 parasite life cycles per year and a resistance allele frequency of 0.01 in 1984. For chloroquine resistance, we modeled positive selection coefficients of s = 0.15 for the *crt-76T* allele and s = 0.13 for the *mdr1-86Y* allele until generation 39 (in 2003), followed by negative selection coefficients of −0.10 and −0.15, respectively (representing fitness costs on these alleles in the absence of drug selection). This was then compared with modeling constant positive selection coefficients. For antifolate resistance, we modeled drug selection coefficients of s = 0.11 for the *dhfr* triple mutant (51I, 59R, 108N) allele and the *dhps 437G* allele throughout the period.

### *P. falciparum* Whole Genome Sequencing and Analysis

Sixty-nine isolates from the coastal area of The Gambia were collected in 2008, processed, and subjected to paired-end short-read sequencing using the Illumina HiSeq platform, following methods previously described [31, 32]. Data from 65 of the isolates were recently used in a genome-wide screen for evidence of balancing selection [32]. The sequence read data from each of these isolates are available from the European Nucleotide Archive, with sample identifiers given in Supplementary Table 1. Short-read sequence data were mapped to the *P. falciparum* 3D7 reference genome (version 3, October 2012 release) using SMALT (http://www.sanger.ac.uk/resources/software/smalt/) run with default parameters. Putative single nucleotide polymorphisms (SNPs) were identified with SAMtools [33] (parameters: samtools mpileup –Q 23 –d 2000 –C50 –ugf) and filtered using the vcfutils.pl script (parameters: varFilter –d 10 –D 2000). Calls were made at each position that passed initial filtering using a majority rules approach, whereby the allele supported by the greatest read count was selected. Positions covered by fewer than 5 reads were marked as missing data for that isolate. SNPs were also filtered in order to remove those located in subtelomeric regions and the hypervariable *var, rifin*, and *stevor* gene families. Following this filtering, 17 of the isolates each had >5000 points of missing SNP data (out of 86 717 identified SNPs). These were dropped from subsequent analysis in order to focus on the majority of isolates (n = 52), which had higher coverage. Individual SNPs were discarded if >3 of these isolates had missing data, and the small amount of remaining missing data were imputed with PHASE 2.1 [34].

### Detection of Signatures of Positive Directional Selection

The standardized integrated haplotype score |iHS| was calculated for each SNP with a minor allele frequency of >0.05 as previously described [35]. This analysis makes an assumption that the majority allele at each SNP position within an isolate comprises a parasite haplotype. The genetic map distance between markers inferred with LDhat 2.2 [36] was measured using a block penalty of 5, 10 million rjMCMC iterations, and a burn-in of 100 000 iterations. Regions under selection were identified by calculating the distance required for the extended haplotype homozygosity (EHH) of high-scoring SNPs (standardized |iHS| >3) to decay to 0.05, with overlapping regions combined into a single window of selection. EHH scores were calculated using the SWEEP program [37].

## RESULTS

### Survey of Drug Resistance Genotypes Over a 25-Year Period

**Figure 1.**
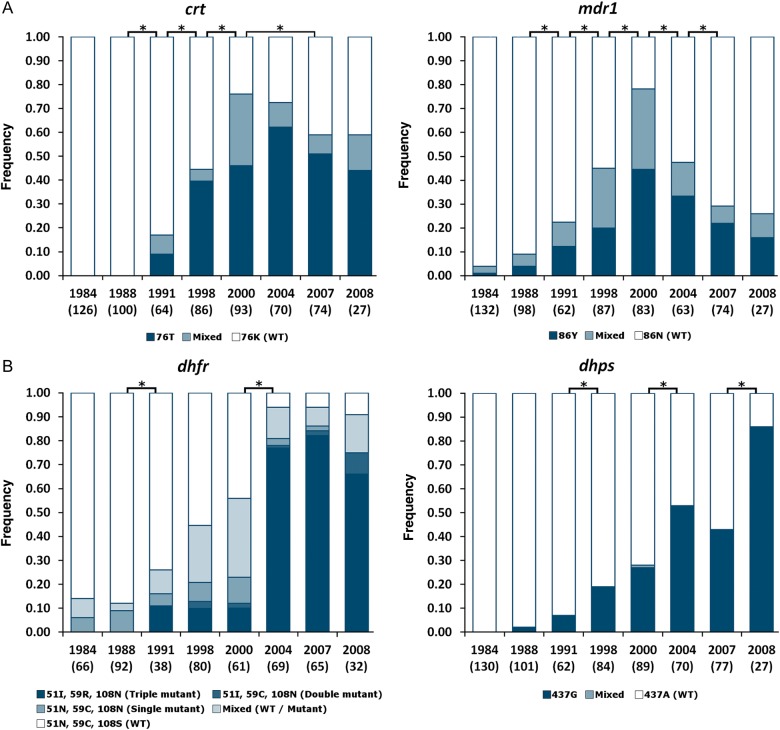
Proportions of *Plasmodium* *falciparum*–infected individuals with each allele at 4 drug resistance loci in 8 surveys conducted in the Farafenni area of The Gambia between 1984 and 2008. *A*, Chloroquine resistance loci (*crt* and *mdr1*). *B*, Antifolate resistance loci (*dhfr* and *dhps*). The numbers of individual infections genotyped for each locus in each year are shown in brackets under each plot. Asterisk indicates significant change (*P* < .05) in the proportions of isolates with each allele between bracketed years (for *dhfr*, the proportion of samples with resistance type at each of the triple positions *dhfr*-51I-59C-108N was compared between years). Abbreviation: WT, wild type.

First, we investigated the historical impact of antimalarial drug pressure in The Gambia by surveying drug resistance polymorphisms in 4 *P. falciparum* genes in samples from 8 time points between 1984 and 2008, with genotyping performed on 668 individuals (Figure 1 and Supplementary Table 2). In the first year, no resistance alleles were detected at 2 of the loci (*pfcrt* and *pfdhps*), while resistance alleles at the other 2 loci (*pfmdr1* and *pfdhfr*) were observed in only very few isolates. Over subsequent survey time points, proportions of isolates with resistance alleles increased progressively and highly significantly (*P* < 10^−7^ for each locus), with the prevalence of chloroquine resistance alleles reaching a peak in 2000 (76% for the *crt* 76T allele, 78% for the *mdr1* 86Y allele). There was a highly significant decline in prevalence of the *mdr1* 86Y allele after 2000 through the end of the survey period (*P* < 10^−7^; Figure 1). Proportions of isolates with antifolate resistance alleles peaked later (94% for *dhfr* alleles in 2007, 86% for the *dhps* 437G allele in 2008; the *dhps* resistance allele 540E that exists in many parts of Africa was not present in any of 623 isolates typed for the causal SNP).

### Direct Evidence of Selection on Drug Resistance Loci

**Figure 2.**
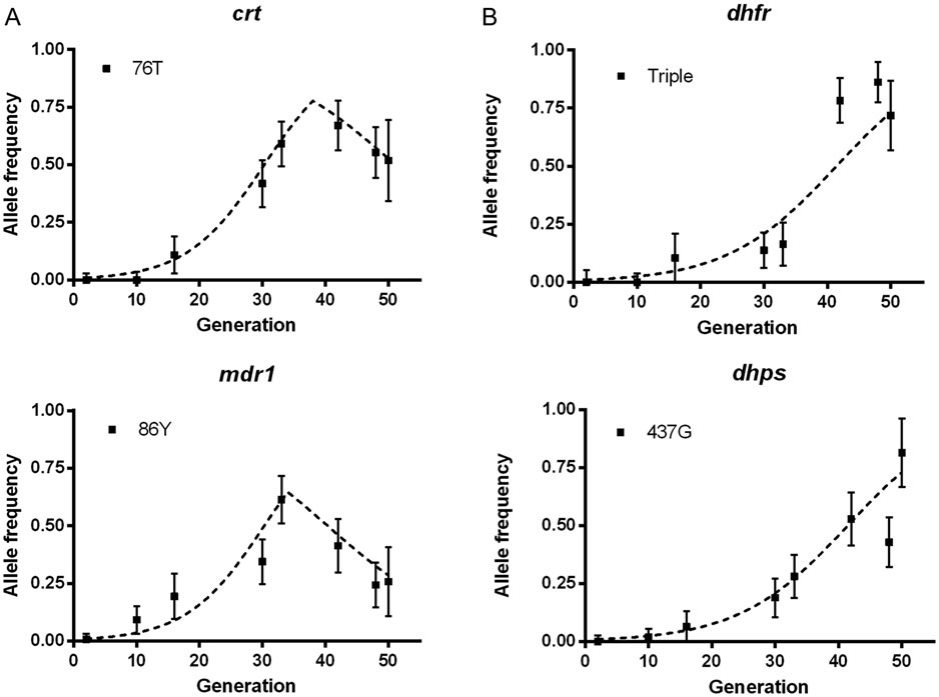
Models of selection at 4 *Plasmodium* *falciparum* drug resistance loci related to allele frequency changes between 1984 and 2008. Allele frequencies (with 95% confidence intervals) of the resistance alleles of (*A*) chloroquine resistance genes *crt* and *mdr1* and (*B*) antifolate resistance genes *dhfr* and *dhfr* are plotted on a time scale, assuming 2 parasite life-cycle generations per year (50 generations from 1984 to 2008). The timing and relative strength of selection at each of the 4 loci was modeled with the following parameters: (*A*) Chloroquine resistance loci: for *crt* 76T positive drug selection coefficient s = 0.15 until generation 39 (in 2003) and s = −0.1 thereafter; for *mdr1* 86Y positive selection s = 0.13 until generation 39 and s = −0.15 thereafter. *B*, Pyrimethamine and sulfadoxine antifolate resistance loci: for *dhfr* triple mutant allele positive selection s = 0.11, and for *dhps* 437G positive selection s = 0.11, throughout the period.

For analysis of population genetic processes, allele frequencies are considered rather than the prevalence of infections with a given allele, as the latter is influenced by the existence of multiple genotypes in some isolates (and would vary over time for any parasite polymorphism when variation in endemicity influences rates of superinfection). Therefore, to estimate allele frequencies, we counted 1 allele at each locus per isolate, randomly sampling in the case of isolates that had mixed genotypes, and estimated the 95% CIs based on numbers of isolates sampled. Figure 2 shows that changes in allele frequencies occurred at different times and rates during the study period. To explore the intensity of selection over time, we compared these data to frequencies generated with a simple model, applying reasonable assumptions of assumed fitness costs and positive selection coefficients that reflect historical therapeutic use. Given that an annual transmission window occurs primarily between August and November, we assumed an average of 2 parasite life-cycle generations per year (Figure 2). The *crt* and *mdr1* chloroquine resistance allele frequencies fit a model in which positive selection continued to operate until sometime between 2000 and 2004 (the model shown sets this as 2003 assuming 2 generations per year; Figure 2*A*). After this time point, assumed fitness costs modeled an allele frequency decline, which was more apparent for *mdr1* than *crt*. The frequency changes at these chloroquine resistance loci clearly did not fit a model of constant positive selection (Supplementary Figure 1). In contrast, changes in frequencies of *dhfr* and *dhps* antifolate resistance alleles closely reflected expectations under continuous positive selection throughout the period studied (Figure 2*B*).

### Signatures of Selection in the Context of Genome-Wide Analysis

**Figure 3.**
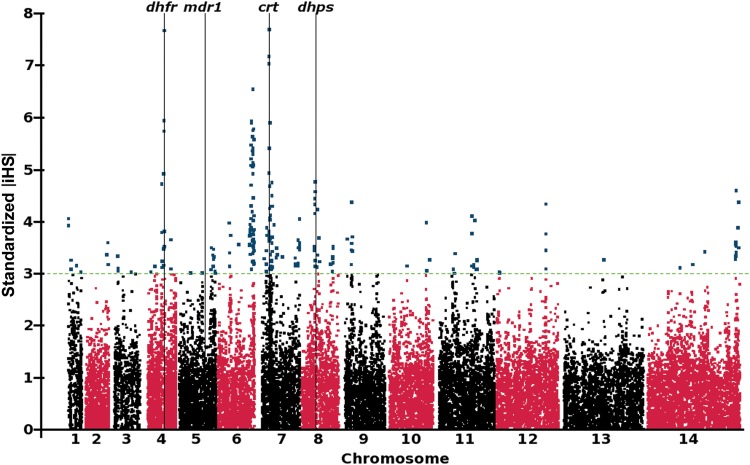
Genome-wide signatures of positive directional selection derived from sequences of a population sample of 52 *Plasmodium* *falciparum* clinical isolates collected in The Gambia in 2008. The standardized integrated haplotype score (|iHS|) is calculated from single nucleotide polymorphism (SNP) data using all SNPs with an allele frequency of >0.05 (in at least 3 individuals). SNPs on individual chromosomes are identified by alternating black/red coloring, with high-scoring SNPs (standardized |iHS| > 3) highlighted in blue. Vertical black lines indicate the position of the 4 drug resistance loci that had been surveyed in the retrospective analysis in this study. Genes within each of the regions of elevated |iHS| supported by at least 2 SNPs are listed in Table 2.

**Figure 4.**
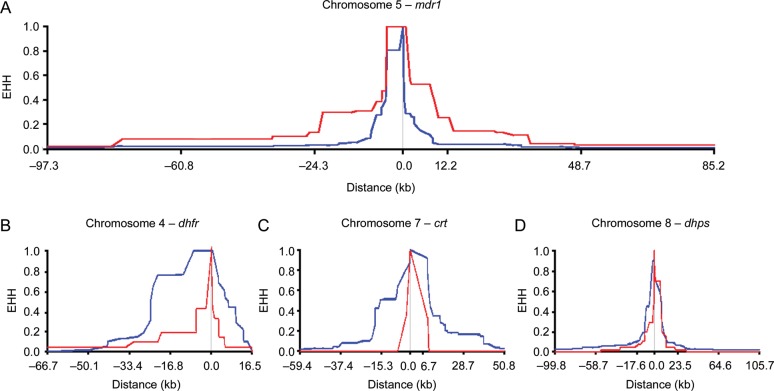
Extended haplotype homozygosity (EHH) decay around each of the 4 drug resistance genes in the sequenced population sample from 2008. Blue indicates EHH decay of the major allele (with allele frequency >0.5 in 2008), red indicates EHH decay of the minor allele. Causative resistance polymorphisms in *dhfr* and *crt* were not called in the single nucleotide polymorphism (SNP) typing applied to the whole genome sequence data. Consequently, these EHH decay plots are presented for the nearest called SNP. *A*, EHH decay centered on *mdr1* codon 86. The resistance allele was present at a frequency of 0.27 and it required 143.8 kb for EHH to decay to a level of 0.05, as opposed to 19.4 kb for the wild-type sensitive allele. *B*, EHH decay for chromosome 4 SNP at position 750 221, the nearest typed SNP to *dhfr* (separated by <2 kb). The major allele was present at a frequency of 0.87 and required 59.4 kb for EHH to decay to 0.05, while the minor allele decayed to this level in 40.0 kb. *C*, EHH decay for chromosome 7 SNP at position 7 399 258, the nearest typed SNP to *crt* codon 76 (separated by 4.4 kb). The major allele was present at a frequency of 0.94 and required 82.3 kb for EHH to decay to 0.05, while the minor allele decayed to this level in 15.9 kb. *D*, EHH decay to 0.05 centered on *dhps* codon 437. The major allele was present at a frequency of 0.77 and required 84.9 kb for EHH to decay to 0.05, while the minor allele decayed to this level in 31.1 kb.

In order to investigate genome-wide signatures of selection, we sampled 69 *P. falciparum* clinical isolates from The Gambia in 2008, and analyzed polymorphism throughout the parasite genome by paired-end short-read sequencing. We identified 86 717 biallelic SNPs in a set of 52 isolates with the most complete data (17 isolates with >5000 untyped SNPs were excluded). Of these, 28 284 SNPs had a minor allele frequency of >0.05, which was informative for long-range haplotype analysis to assess the impact of selection. Calculation of the |iHS| identified 22 discrete genomic regions containing a candidate signature of selection, as defined by 2 or more SNPs with standardized |iHS| >3 (Figure 3 and Table 2). Three of the top 4 signatures, as defined by the number of supporting SNPs with high |iHS| values, contained the *dhfr*, *crt*, and *dhps* drug resistance genes (Figure 3). We did not detect any comparable signature around the *mdr1* gene (locus Pf3D7_0523000), although EHH was associated with the *mdr1* 86Y resistance allele (Figure 4), which had declined in frequency by 2008. In association with this resistance allele, it took a distance of 143.8 kb for EHH to decay to a level of 0.05, as opposed to 19.4 kb for the wild-type sensitive allele. For the other drug resistance genes, long haplotypes were associated with resistance alleles present at high frequency in 2008 (Figure 4), as expected for loci under very recent positive selection and contributing to the strong |iHS| signatures at these loci.

**Table 2.** Genomic Windows Containing Signatures of Recent Positive Selection as Identified by Genome Wide Standardized Integrated Haplotype Score Scan

Chromosome	Start Position (kb)	End Position (kb)	Region Size (kb)	Number of Supporting Single Nucleotide Polymorphisms	Number of Genes in the Region	Genes Within Region
1	94.7	338.3	243.6	4	63	PF3D7_0102100 – PF3D7_0108300
2	763.8	829.9	66.1	3	20	PF3D7_0218700 – PF3D7_0220600
3	120.4	249.7	129.3	4	30	PF3D7_0302200 – PF3D7_0305100
4	274.5	289.6	15.1	2	3	PF3D7_0405100 – PF3D7_0405300
4	***585.1***	***766.1***	***181.0***	***14***	***46***	***PF3D7_0413000 – PF3D7_0417400***
4	966.5	993.0	26.5	3	6	PF3D7_0421200 – PF3D7_0421700
5	1129.4	1323.6	194.2	11	58	PF3D7_0527100 – PF3D7_0532700
6	340.7	528.5	187.8	4	49	PF3D7_0608100 – PF3D7_0612800
6	1031.1	1301.9	270.8	65	59	PF3D7_0625300 – PF3D7_0631100
7	***240.8***	***520.0***	***279.2***	***53***	***70***	***PF3D7_0704800 – PF3D7_0711700***
7	589.6	696.4	106.8	4	30	PF3D7_0712900 – PF3D7_0715800
7	1253.1	1351.5	98.4	4	21	PF3D7_0729400 – PF3D7_0731300
7	1380.5	1406.7	26.2	4	8	PF3D7_0731800 – PF3D7_0732500
7	1427.6	1437.7	10.1	4	0	…
8	***427.6***	***708.9***	***281.3***	***18***	***66***	***PF3D7_0808500 – PF3D7_0814900***
8	974.3	1193.0	218.7	12	59	PF3D7_0821800 – PF3D7_0827600
9	144.6	371.7	227.1	8	51	PF3D7_0903300 – PF3D7_0908000
11	518.9	775.4	256.5	3	77	PF3D7_1113300 – PF3D7_1120600
11	1197.7	1477.5	279.8	10	70	PF3D7_1131100 – PF3D7_1137600
12	57.0	178.6	121.6	2	29	PF3D7_1200700 – PF3D7_1203400
12	1709.0	1745.0	36.0	6	6	PF3D7_1240400 – PF3D7_1240900
14	3050.6	3203.2	152.6	10	34	PF3D7_1474400 – PF3D7_1477700

Regions on chromosomes 4, 7, and 8, which encompass the drug resistance genes *dhfr* (Pf3D7_0417200), *crt* (Pf3D7_0709000), and *dhps* (Pf3D7_0810800), respectively, are in bold italics.

The scan shown in Figure 3 clearly identifies other genomic loci under recent positive selection (Table 2). An exceptionally strong signature, supported by 65 SNPs with standardized |iHS| > 3, implicates a large region of approximately 271 kb on chromosome 6, which covers 59 genes. Strong directional selection on the region has been previously inferred by analysis of genome-wide sequence data from a population in Senegal [38] and SNP microarray data from a combined population sample from The Gambia and Senegal [39]. The identification of a strong |iHS| signature in the region of chromosome 11 containing the *ama1* antigen gene (locus Pf3D7_1133400) is also notable, as this gene has been previously implicated to be under balancing selection [32] and positive directional selection [39].

## DISCUSSION

We have described allele frequency trajectories over time in relation to known selective causes and then evaluated signatures of directional selection in a genome-wide analysis. The temporal changes in *P. falciparum* drug resistance allele frequencies over the 25-year period were consistent with the history of varying antimalarial drug use in the Gambian population during this period. Resistance-associated alleles were virtually absent from the earliest survey in 1984, as expected from results of a large survey in this area in 1982 that indicated no resistance of *P. falciparum* to chloroquine, pyrimethamine, or dapsone (sulfadoxine was not used in the country at that time) [40]. Chloroquine was the first-line therapy during this period, so resistance to this drug emerged first [41, 42], and we show progressive increases in allele frequencies of *crt*-76T and *mdr1*-86Y in all subsequent surveys until 2000. Although sulfadoxine–pyrimethamine remained officially as second-line treatment until 2004, its use for first-line treatment is considered to have been widespread for a long time before this, given the apparent failure of chloroquine [43]; the continuous rise in frequency of antifolate drug resistance alleles *dhfr*-51I-59C-108N and *dhps*-437G reflects this.

The temporal allele frequency change data at each of these 4 loci closely fit with expectations from a very simple selective model that had only a few parameters, incorporating constant fitness costs. We excluded other complexities, such as seasonal differences in selection [44], differences in fitness costs, times of introduction of different alleles, and possible epistatic interactions among alleles at different loci [29], which could contribute to a more detailed model if the relevant parameters were known [45]. Estimated selection coefficients depend on the number of parasite life-cycle generations per year. Our estimates, which are based on either 2 or 3 generations per year, are similar to those from studies elsewhere in Africa [15, 18], except where an exceptionally large number of generations per year was assumed [13]. The timing of the changes in selection are consistent with perceptions that the actual use of chloroquine declined markedly in response to widely perceived therapeutic failure, while sulfadoxine–pyrimethamine continued to be used although chloroquine alone was recommended as first-line treatment until 2004 and in combination with sulfadoxine–pyrimethamine until 2008.

In the genome-wide analysis of samples from 2008, 3 of these drug resistance loci (*crt*, *dhfr*, and *dhps*) were within the top 4 |iHS| signatures, independently indicating recent strong positive selection. There was power to detect these signatures by sampling at that single time point, as the resistance-associated alleles remained common in the population until the population sample was taken for genome-wide sequencing in 2008, even though the frequency of the *crt* resistance allele may have begun to decline by that time. In contrast, the fact that the *mdr1* gene region was not identified from the genome-wide scan is likely to be due to a lower resistance allele frequency, which had declined very significantly between 2000 and 2008. Although the *mdr1* haplotype homozygosity was greater for the resistant allele than for the sensitive allele, it was not significantly greater than the possible range expected under neutrality, given the relatively low allele frequency, and thus a transient genomic signature might have been missed by sampling too late. Two other studies from this area of West Africa have also not detected clear signals of selection at this locus by |iHS| analysis [38, 39]. One of these involved analysis on Senegalese samples taken during a period of chloroquine and amodiaquine use in that country [38]. However, a separate analysis of a subsample of the Senegalese population sample did show a selective sweep signature at the *mdr1* locus [46], supporting the idea that exact location or timing of sampling may have a major effect in this situation. We cannot exclude the possibility that there has been selection on multiple preexisting alleles of *mdr1*, as was the case in a soft selective sweep on alleles containing copy number amplifications of the *mdr1* gene in Southeast Asia [3], although copy number variants at this locus are extremely rare in West Africa [47].

Of the other genomic loci identified as most likely to have been under recent directional selection, a few were also identified recently from independent analysis of samples from The Gambia and Senegal using an Affymetrix custom SNP array [39] and sequence-based analysis of parasites from Senegal [38]. The selective signature on chromosome 6 was previously associated with parasites that were pyrimethamine resistant in Senegal [38], but the test could not identify the exact gene under selection. Two of the genes within the locus (PF3D7_062800 and PF3D7_0630700) have proposed roles in folate metabolism pathways, so it could be speculated that selection operates on compensatory mutant alleles that offset loss of dihydrofolate reductase function from partial inhibition by subtherapeutic levels of pyrimethamine. However, due to the wide selection window and limitations of haplotype-based methods, we cannot exclude the possibility that other genes within the locus are the targets of selection. We consider it likely that a strong signature in chromosome 11 may be due to recent immune selection of a novel allele of *ama1* added to a broad repertoire of alleles under balancing selection [39]. The causes of selection in these cases deserve detailed investigation, as there are only a few loci genome wide that show such strong local selective sweeps. The likelihood that we have already detected most of the genomic loci affected by such strong recent selection has 2 main implications. First, if future drug resistance alleles are under similarly strong selection, in most cases, it should be possible to detect the emerging selective sweeps in genome-wide scans, and efforts toward such surveillance should be increased in Africa where the burden of malaria remains highest and the public health significance of drug resistance is greatest. Second, other tests are needed to detect more moderate or complex processes of positive selection that dominate in evolution generally and that will pertain to important phenotypes other than drug resistance.

## Supplementary Data

Supplementary materials are available at *The Journal of Infectious Diseases* online (http://jid.oxfordjournals.org/). Supplementary materials consist of data provided by the author that are published to benefit the reader. The posted materials are not copyedited. The contents of all supplementary data are the sole responsibility of the authors. Questions or messages regarding errors should be addressed to the author.

Supplementary Data
