# Supplementary material for: Changes in Malaria Parasite Drug Resistance in an Endemic Population Over a 25-Year Period With Resulting Genomic Evidence of Selection
Source: J Infect Dis. 2013 Nov 21;209(7):1126–35. doi: 10.1093/infdis/jit618 (PMC3952670; doi:10.1093/infdis/jit618)
Supplement: Supplementary Data [file supp_209_7_1126__index.html]

Changes in malaria parasite drug resistance in an endemic population over a 25-year period with resulting genomic evidence of selection — Changes in Malaria Parasite Drug Resistance in an Endemic Population Over a 25-Year Period With Resulting Genomic Evidence of Selection — Changes in Malaria Parasite Drug Resistance in an Endemic Population Over a 25-Year Period With Resulting Genomic Evidence of Selection — Supplementary Data 

# Changes in Malaria Parasite Drug Resistance in an Endemic Population Over a 25-Year Period With Resulting Genomic Evidence of Selection

## Supplementary Data

Supplementary Data

**Files in this Data Supplement:**

- Supplementary Figure 1 - pdf file
- Supplementary Table 1 - docx file
- Supplementary Table 2 - docx file
