# Supplementary material for: Changes in Malaria Parasite Drug Resistance in an Endemic Population Over a 25-Year Period With Resulting Genomic Evidence of Selection
Source: J Infect Dis. 2013 Nov 21;209(7):1126–35. doi: 10.1093/infdis/jit618 (PMC3952670; doi:10.1093/infdis/jit618)
Supplement: Supplementary Data [file supp_jit618_jit618supp_fig1.pdf]

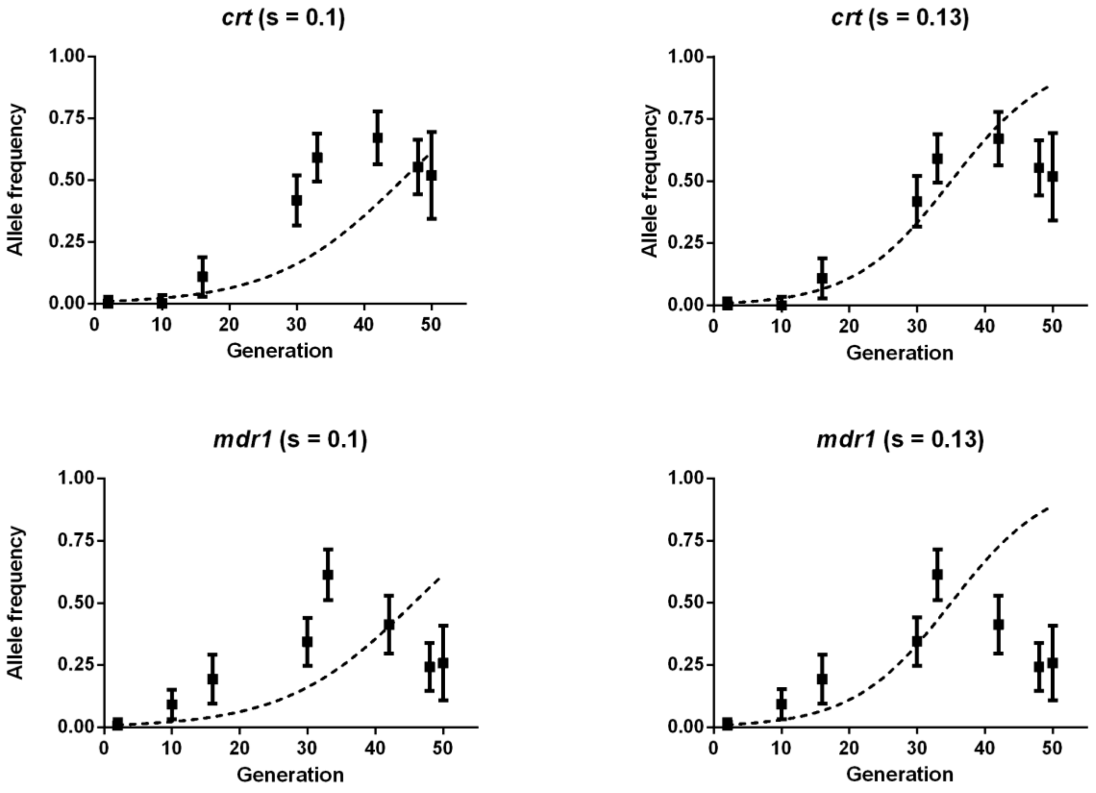

Supplementary Figure S1. Models of constant positive selection do not fit the temporal pattern of allele frequency change at chloroquine resistance loci. Allele frequencies (with 95%CI) of the resistance alleles of genes *crt* (codon 76T) are shown on the top row, and *mdr1* (codon 86Y) on the bottom row*,* plotted on a time scale assuming 2 parasite life cycle generations per year (50 generations from 1984 to 2008). Models of constant positive selection are shown with dashed lines for selection coefficients of s = 0.10 (left column) and s = 0.13 (right column).
