# Supplementary material for: Changes in Malaria Parasite Drug Resistance in an Endemic Population Over a 25-Year Period With Resulting Genomic Evidence of Selection
Source: J Infect Dis. 2013 Nov 21;209(7):1126–35. doi: 10.1093/infdis/jit618 (PMC3952670; doi:10.1093/infdis/jit618)
Supplement: Supplementary Data [file supp_jit618_jit618supp_table1.docx]

Supplementary Table S1. Summary of whole-genome sequence reads, mapping and SNP calling for 69 Gambia clinical isolates sampled in 2008.

| Sample_Seq_ID | ENA accession no. | Number of mapped reads | Average read  depth coverage  (genome wide, x) | Average read  depth coverage  (coding regions, x) |
| --- | --- | --- | --- | --- |
| PA0007-C | ERS010116 | 27297504 | 69.7 | 101.2 |
| PA0008-C | ERS010126 | 33288726 | 88.5 | 120.5 |
| PA0011-C | ERS010043 | 48787238 | 136.7 | 154.6 |
| PA0012-C | ERS010127 | 38300312 | 87.9 | 121.7 |
| PA0015-C | ERS010044 | 12955162 | 36.4 | 40.8 |
| PA0016-C | ERS010045 | 37924896 | 107.4 | 121.4 |
| PA0017-C | ERS010128 | 67872370 | 177.2 | 243.6 |
| PA0018-C | ERS010046 | 6829326 | 19.8 | 21.2 |
| PA0020-C | ERS010047 | 51636442 | 149.3 | 164.2 |
| PA0021-C | ERS010048 | 40713586 | 117.9 | 130.4 |
| PA0022-C | ERS010129 | 26378200 | 67.6 | 95.6 |
| PA0026-C | ERS011006 | 3646350 | 9.5 | 11.2 |
| PA0027-C | ERS010049 | 40572206 | 119.2 | 131.0 |
| PA0029-C | ERS010038 | 49381786 | 138.3 | 153.8 |
| PA0030-C | ERS009726 | 58666268 | 154.1 | 173.0 |
| PA0032-C | ERS010130 | 67551598 | 169.3 | 236.1 |
| PA0034-C | ERS010117 | 38199296 | 75.4 | 103.9 |
| PA0035-C | ERS010039 | 126480592 | 344.3 | 385.5 |
| PA0036-C | ERS010040 | 42413982 | 118.3 | 135.5 |
| PA0037-C | ERS010041 | 33650826 | 87.4 | 99.6 |
| PA0038-C | ERS009729 | 59920658 | 162.0 | 185.9 |
| PA0039-C | ERS009966 | 54199910 | 144.0 | 200.6 |
| PA0040-C | ERS010118 | 30509798 | 80.2 | 111.9 |
| PA0041-C | ERS010119 | 62282512 | 137.1 | 197.7 |
| PA0042-C | ERS010120 | 58032108 | 134.7 | 188.9 |
| PA0044-C | ERS009731 | 51705254 | 142.2 | 158.9 |
| PA0045-C | ERS009732 | 36927886 | 98.2 | 107.8 |
| PA0046-C | ERS009733 | 33507866 | 94.7 | 109.5 |
| PA0047-C | ERS010113 | 5542870 | 13.7 | 16.2 |
| PA0049-C | ERS010071 | 57188406 | 158.1 | 204.1 |
| PA0050-C | ERS009669 | 25949700 | 70.0 | 88.1 |
| PA0051-C | ERS011004 | 6871340 | 18.7 | 27.7 |
| PA0052-C | ERS010070 | 64988842 | 171.7 | 239.4 |
| PA0053-C | ERS009967 | 59879784 | 158.4 | 211.5 |
| PA0054-C | ERS011013 | 4584238 | 12.6 | 18.8 |
| PA0056-C | ERS011002 | 3988770 | 9.5 | 13.7 |
| PA0057-C | ERS011016 | 81141416 | 227.7 | 292.8 |
| PA0059-C | ERS011009 | 2703168 | 6.8 | 9.2 |
| PA0060-C | ERS009674 | 13900486 | 37.6 | 46.9 |
| PA0061-C | ERS009670 | 19065168 | 46.4 | 57.9 |
| PA0063-C | ERS009671 | 12683326 | 25.9 | 28.1 |
| PA0064-C | ERS011012 | 3076826 | 8.2 | 11.2 |
| PA0065-C | ERS010079 | 42756702 | 115.1 | 141.7 |
| PA0066-C | ERS009679 | 44284944 | 93.7 | 111.7 |
| PA0067-C | ERS011019 | 67260170 | 197.1 | 260.8 |
| PA0068-C | ERS009970 | 63391822 | 152.3 | 212.6 |
| PA0069-C | ERS009971 | 62420686 | 147.0 | 203.2 |
| PA0071-C | ERS010078 | 36863976 | 80.7 | 107.0 |
| PA0073-C | ERS010076 | 9716191 | 23.9 | 31.4 |
| PA0074-C | ERS010069 | 33906686 | 94.2 | 125.9 |
| PA0075-C | ERS010074 | 46703452 | 123.7 | 152.9 |
| PA0078-C | ERS011014 | 61746106 | 82.6 | 105.8 |
| PA0079-C | ERS011008 | 2888376 | 7.9 | 11.4 |
| PA0081-C | ERS009676 | 50798934 | 133.1 | 174.5 |
| PA0084-C | ERS009972 | 50759990 | 127.3 | 175.7 |
| PA0085-C | ERS009672 | 11307982 | 30.4 | 39.7 |
| PA0087-C | ERS011011 | 7592826 | 20.8 | 25.8 |
| PA0089-C | ERS011010 | 4485628 | 12.1 | 16.8 |
| PA0091-C | ERS009973 | 57431350 | 144.9 | 200.9 |
| PA0092-C | ERS010068 | 23929924 | 57.1 | 75.8 |
| PA0093-C | ERS010077 | 43477528 | 116.0 | 143.5 |
| PA0094-C | ERS011005 | 3787858 | 10.7 | 13.1 |
| PA0098-C | ERS011018 | 34891156 | 93.6 | 106.6 |
| PA0099-C | ERS010080 | 49355872 | 129.3 | 164.5 |
| PA0100-C | ERS011020 | 65246982 | 184.3 | 249.9 |
| PA0101-C | ERS009975 | 7790984 | 205.1 | 292.0 |
| PA0102-C | ERS009673 | 39793874 | 89.2 | 105.5 |
| PA0104-C | ERS011015 | 84247654 | 239.7 | 272.7 |
