# Supplementary material for: Changes in Malaria Parasite Drug Resistance in an Endemic Population Over a 25-Year Period With Resulting Genomic Evidence of Selection
Source: J Infect Dis. 2013 Nov 21;209(7):1126–35. doi: 10.1093/infdis/jit618 (PMC3952670; doi:10.1093/infdis/jit618)
Supplement: Supplementary Data [file supp_jit618_jit618supp_table2.docx]

Supplementary Table S2. Numbers of isolates positive for each of the drug resistance alleles in each year sampled.

| Gene | Allele | Year | | | | | | | |
| --- | --- | --- | --- | --- | --- | --- | --- | --- | --- |
|  |  | 1984 | 1988 | 1991 | 1998 | 2000 | 2004 | 2007 | 2008 |
| *dhfr* | 51N, 59C, 108S | 57 | 81 | 28 | 45 | 27 | 4 | 4 | 3 |
|  | (WT sensitive only) |  |  |  |  |  |  |  |  |
|  | 51N, 59C, 108N  (Single mutant only) | 4 | 8 | 2 | 6 | 7 | 2 | 1 | 0 |
|  | 51I, 59C, 108N  (Double mutant only) | 0 | 0 | 0 | 2 | 1 | 1 | 1 | 3 |
|  | 51I, 59R, 108N  (Triple mutant only) | 0 | 0 | 4 | 8 | 6 | 53 | 54 | 21 |
|  | Mixed (WT / Mutant) | 5 | 3 | 4 | 19 | 20 | 9 | 5 | 5 |
|  |  |  |  |  |  |  |  |  |  |
| *mdr1* | 86N (WT) | 127 | 89 | 47 | 48 | 18 | 33 | 50 | 23 |
|  | Mixed | 4 | 5 | 6 | 22 | 28 | 9 | 5 | 3 |
|  | 86Y | 1 | 4 | 9 | 17 | 37 | 21 | 16 | 5 |
|  |  |  |  |  |  |  |  |  |  |
| *crt* | 76K (WT) | 126 | 100 | 53 | 48 | 22 | 19 | 30 | 11 |
|  | Mixed | 0 | 0 | 5 | 4 | 28 | 8 | 6 | 4 |
|  | 76T | 0 | 0 | 6 | 34 | 43 | 43 | 38 | 12 |
|  |  |  |  |  |  |  |  |  |  |
| *dhps* | 437A (WT) | 130 | 98 | 58 | 68 | 64 | 33 | 44 | 3 |
|  | Mixed | 0 | 0 | 0 | 0 | 1 | 0 | 0 | 0 |
|  | 437G | 0 | 2 | 4 | 16 | 24 | 37 | 33 | 19 |
|  |  |  |  |  |  |  |  |  |  |
